# Nm23-H1 is involved in the repair of ionizing radiation-induced DNA double-strand breaks in the A549 lung cancer cell line

**DOI:** 10.1186/s12885-018-4592-2

**Published:** 2018-07-03

**Authors:** Ya Sheng, Mingfang Xu, Chongyi Li, Yanli Xiong, Yi Yang, Xunjie Kuang, Dong Wang, Xueqin Yang

**Affiliations:** Cancer Center, Daping Hospital and Research Institute of Surgery, Army Military Medical University, No.10 Changjiang Zhi lu, Daping Yuzhong District, Chongqing, 400042 China

**Keywords:** Nm23-H1, Double-strand break repair, Lung cancer

## Abstract

**Background:**

Although originally identified as a putative metastasis suppressor, increasing studies have confirmed a possible role for Nm23-H1 in DNA repair, through the base excision repair and nucleotide excision repair pathways. In this study, we explored whether Nm23-H1 was also involved in double-strand break repair (DSBR).

**Methods and results:**

We constructed a stable A549-shNm23-H1 cell line with doxycycline-regulated expression of Nm23-H1, and a A549-nNm23-H1 cell line that over expressed a nucleus-localized version of Nm23-H1. Results from both lines confirmed that Nm23-H1 participated in the repair of double-strand breaks induced by X-rays, using Comet and γ-H2AX foci assays. Subsequent studies showed that Nm23-H1 activated the phosphorylation of checkpoint-related proteins including ATM serine/threonine kinase (on S1981), tumor protein p53 (on S15), and checkpoint kinase 2 (Chk2) (on T68). We also detected interactions between Nm23-H1 and the MRE11-RAD50-NBS1 (MRN) complex, as well as Ku80. Moreover, NBS1 and Ku80 levels were comparably higher in Nm23-H1 overexpressing cells than in control cells (*t* = 14.462, *p* < 0.001 and *t* = 5.347, *p* = 0.006, respectively). As Ku80 is the keystone of the non-homologous end joining (NHEJ) pathway, we speculate that Nm23-H1 promotes DSBR through NHEJ.

**Conclusions:**

The results indicate that Nm23-H1 participates in multiple steps of DSBR.

## Background

Nm23-H1 is a multifunctional enzyme with decreased expression in certain highly metastatic cell lines and tumors, and was initially identified as a putative metastasis suppressor [1]. It possesses nucleoside diphosphate kinase (NDPK) activity, which maintains the intracellular nucleotide balance and is required for DNA synthesis [2]. It also has 3′-5′ exonuclease activity [3], which executes the stepwise excision of damaged or mispaired nucleotides during DNA replication and repair [4]. Because of this, Nm23-H1 is thought to play a role in DNA repair. However, the molecular mechanisms by which Nm23-H1 functions as a DNA repair gene remain unclear.

DNA damage repair takes three forms, namely, base excision repair (BER), nucleotide excision repair (NER), and double strand break repair (DSBR). The first evidence in support of a DNA repair function for Nm23-H1 was obtained in *Saccharomyces cerevisiae*, in which ablation of the Nm23-H1 homolog YNK1 resulted in delayed and error-prone repair of DNA lesions induced by ultraviolet radiation and the DNA topoisomerase II inhibitor etoposide [5]. We reported the second in a previous study, in which we observed that the nuclear localization of Nm23-H1 gradually increased 24 and 48 h after X-ray irradiation and demonstrated that Nm23-H1 was involved in BER, interacting with the BER protein apurinic/apyrimidinic endodeoxyribonuclease 1(APE1) [6]. At almost the same time, Jarrett et al. reported that human melanoma cell lines with coordinately low expression of Nm23-H1 and Nm23-H2 repaired UV-induced 6–4 photoproducts and other DNA polymerase-blocking lesions at a slower rate, and that the kinetics of repair were accelerated significantly upon forced expression of Nm23-H1, through enhanced NER [7]. However, both etoposide and X-rays mainly induce double-strand breaks (DSBs). Thus, in this study, we explored whether Nm23-H1 was also involved in DSBR. In addition, as previous studies have investigated the effects of cytosolic Nm23-H1 on DNA repair, which occurs mainly in the nucleus, nuclear Nm23-H1 was introduced for the first time to investigate the mechanism of its involvement in DSBR.

## Methods

### Vector construction

Vectors were constructed by using standard cloning procedures as before [8]. Dox-regulated vector system of conditional suppressing the expression of Nm23-H1 was constructed. The Nm23-H1 was suppressed only when addition with doxycycline (DOX). In briefly, Nm23-H1-shRNA was introduced into Lentis-BiD-tetO-H1-SFFV-GFP vector and driven by H1 promoter. Lentis-BiD-tetO-H1-SFFV-GFP vector also carried the green fluorescent protein (GFP) reporter gene driven by the SFFV promoter and a tetracycline operator. The targeted interference sequence of Nm23-H1 is CGTACCTTCATTGCGATCAA.

We also constructed a vector that over expressed Nm23-H1 with nuclear located sequence (NLS) which can introduce Nm23-H1 into nucleus. Nm23-H1 amplification fragment was from the pEGFP-Nm23-H1 using the upper primer with NLS and cloned into pLentis-CMV-IRES2-PURO vector, downstream of the CMV promoter. Upper primer: 5′-TTAGGATCCACCATGAAGCG ACCTGCCGCCACAAAGAAGGCTGGACAGGCTAAGAAGAAGAAAATGGCCAACTGTG AG-3′;Downstream primers:5′-GCACTCGAGTTAAGCATAATCTGGAACATCATATGG ATATTCATAGATCCAGTTCT-3′.

### Cell transfection

A549 (human lung cancer cell line,3111C0001CCC000002) and 293 T (Human embryonic kidney T cell line,3111C0001CCC000010) were obtained from the National Infrastructure of Cell Line Resource (Beijing, China). Cell culture was carried out under 5% CO_2_ and 37 °C using DMEM medium with 10% FBS and non-essential amino acids. According to standard protocols, all recombinant lentiviruses were generated by transit transfection of 293 T cells. Briefly, subconfluent 293 T cells were cotransfected with 1.5 μg of pMD2.G, 4.5μg of psPAX2, and 6 μg of pLentis-nm23 or FCBsdKRABW (control) by calcium phosphate precipitation. After cell culture for 16 h, medium was changed, and 24 h later, recombinant lentivirus vectors were harvested. For construction of A549-shNm23-H1 and A549-nNm23-H1 cell lines, A549 cells were placed on 24-well plate (2×10^4^ cells/well). After cell culture for 16 h, medium containing recombinant lentivirus vectors was added. Then 1 μg/ml puromycin was added for selecting the positive clone 24 h later. To analyze the regulation of doxycycline (DOX) on Nm23-H1, DOX was added to the transduced cells at a final concentration of 5 μg/ml. Protein level was detected by Western blot and the nuclear location was detected by confocal microscopy, which will describe later.

### Cell irradiation and colony formation assay

For X-irradiation, samples were cultured in 25 cm^2^ flasks until they reached 75% fullness, and were then irradiated at room temperature with an Precise Linear Accelerator (Elekta, 8 MV) using different doses. The error of exposure dose was calculated to be within 15%. More details were performed as described previously [6].

For the colony formation assay, cells after irradiation were cultured on a 6-well plate at 500 cells per well for 10–14 days, and the colonies were counted after being sequentially fixed with methanol and stained with 0.5% crystal violet solution.

### Immunofluorescence staining and confocal microscopy

Cells after irradiation were digested with trypsin, fixed with paraformaldehyde 2% in phosphate buffer solution (PBS), permeabilized with a solution of 0.5% of Triton X in PBS, and blocked with 1 ml of 0.4% BSA in PBS. Thereafter, the cells were kept at 4 °C for a maximum of a week, and then the following steps are carried out at room temperature. After washing, cells were resuspended in 100 μl of primary antibody solution (mouse monoclonal, anti-H2AX Ser139 antibody and anti-Nm23-H1 antibody) and incubated for 1 h. Then the cells were washed again, resuspended in 100 μl of secondary antibody (1:400; Alexa 488 and Alexa Fluor555 goat anti-mouse F(ab)2 conjugate) and incubated in the dark for 45 min. Finally, the cells was incubated with 1 ml of DAPI (1:1000) for DNA staining at least 15 min in the dark. The fluorescence intensity and the foci numbers of the cells was measured at the confocal microscopy. One huandred cells from each experiment were randomly selected for counting γ-H2AX foci present in each nucleus and more than 10 foci per nucleus are defined as positive cells. For more accurate comparisons, cells in the same experiments were stained and measured on the same day [9]. Experiments were performed in three individual replicates.

### Comet assay

Comet assay was carried out with reference to the method of An J et al. In briefly, After 8 Gy X rays irradiated and cultured for 0~ 8 h, cells were collected and mixed with low melting point agarose at 37 °C. This mixture was placed on a previously formed layer of 0.5% normal melting point (NMP) agarose on a slide, covered with a cover slip, and incubated at 4 °C until the solidification state. Then, the cover slip was removed, added with another layer of NMP agarose on the top, and repeat above steps until the mixture was solidified again. The slides were placed in chilled neutral lysis solution for electrophoresis. After that, slides were gently washed with neutralization buffer, then stained with ethidium bromide and observed under a fluorescence microscope. DNA damage was expressed as the tail moment, which combined tail length of the comet and the proportion of DNA migrating into the tail [10]. The images were analyzed using OpenComet software (v1.3.1). Experiments were performed in three individual replicates.

### Western blotting, co-immunoprecipitation, and antibodies

Total cellular protein extracted by lysis buffer (250 mM NaCl, 0.1% Nonidet P-40,50 mM HEPES (pH 7.6), and 5 mM EDTA). Proteins were separated by sodium dodecyl sulfate-polyacrylamide gel electrophoresis (SDS-PAGE) [11]. Western blotting was performed as described previously [6, 12].

For co-immunoprecipitation, which has been described as before [6], the cell protein (200 μg) was incubated with 5 μg of anti-Nm23-H1 monoclonal antibody and rotate for 12 h at 4 °C. Protein G agarose beads were then added and agitated for 4 h at 4 °C. The immunoprecipitated material was washed and centrifugated for three times in ProFound lysis buffer to remove the unbound substances. The final pellet was boiled in SDS loading buffer and the agarose beads were removed from the precipitated proteins by centrifugation. The supernatant was then subjected to Western blotting using NBS1, RAD50, MRE11, Ku80 and DNA-PKcs antibody for immunoprecipitation and normal rabbit IgG was used for control. Experiments were performed in three individual replicates.

Most of the antibodies and their phosphorylated antibodies were purchased from Cell Signaling Technology, Inc., (Danvers, MA, USA), except for the rabbit anti-nm23-H1 antibody (Santa Cruz), mouse anti-nm23 antibody (nm301, Calbiochem), and Ku80 (Abcam).

### Statistical analysis

Statistical and mathematical analyses of the data were conducted using the SPSS 17.0 software. Quantitative data were obtained from three independent experiments and expressed as mean ± SD values. Statistical differences between two groups were determined using Student’s t test. *P* values were two-sided, and those < 0.05 were considered as statistically significant [6, 12].

## Results

### Nm23-H1 promotes the repair of X-ray-induced DSBs

**Fig. 1:**
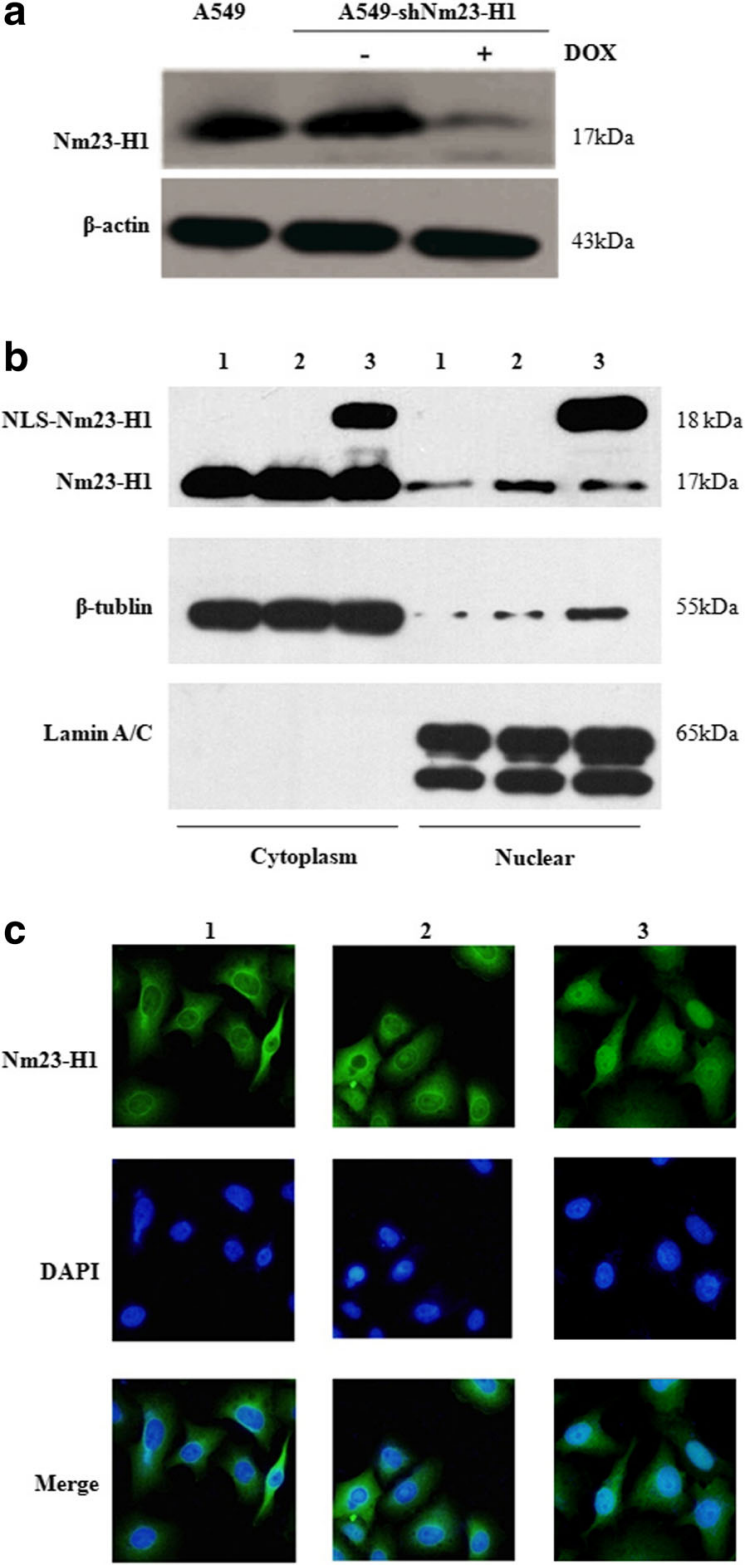
The expression and location of Nm23-H1 in A549-shNm23-H1 cell line and A549-nNm23-H1 cell line. A549-shNm23-H1 cell line was a Dox-regulated vector system of conditional suppressing the expression of Nm23-H1, and the Nm23-H1 expression was suppressed only when doxycycline was added. A549-nNm23-H1 cell line was constructed for over expressed Nm23-H1 with nuclear located sequence (NLS) which can introduce Nm23-H1 into nucleus. **a** The expression of Nm23-H1 in A549-shNm23-H1 cell line detected by Western blot. **b** The expression and location of Nm23-H1 in A549-nNm23-H1 cell line detected by Western blot NLS-Nm23-H1 is the constructed protein(18 kDa) and Nm23-H1 is the endogenous protein (17 kDa). 1 A549; 2 A549-vector; 3 A549-nNm23-H1. **c** The expression and location of Nm23-H1 detected by confocal microscopy in A549-nNm23-H1 cell line. The Nm23-H1 protein is stained with green fluorescent and the nucleus is stained with blue fluorescent . 1 A549; 2 A549-vector; 3 A549-nNm23-H1

To investigate the role and mechanism of Nm23-H1 in DSBR, we constructed a stable A549-shNm23-H1 cell line with doxycycline-regulated expression of Nm23-H1.We also constructed a stable A549-nNm23-H1 cell line that overexpressed Nm23-H1 and a nuclear localization sequence (NLS) to introduce Nm23-H1 into the nucleus, which would be beneficial to the follow-up experiment on the interaction of protein in the nucleus. The Nm23-H1 protein was markedly depleted in the A549-shNm23-H1 cells and successfully localized to the nuclei of A549-nNm23-H1 cells (Fig. 1).

**Fig. 2:**
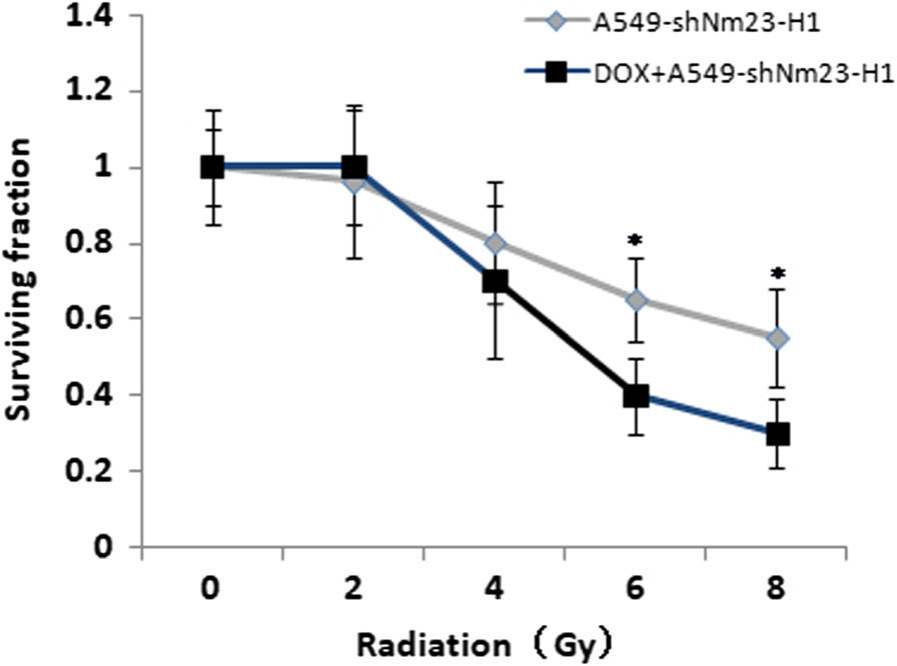
Colony formation assay of the cells after irradiation. Cells were irradiated with different doses (0Gy, 2Gy, 4Gy, 6Gy and 8Gy) and then were cultured for 10–14 days. Colonies were counted after fixing and staining (*: *p* < 0.05)

**Fig. 3:**
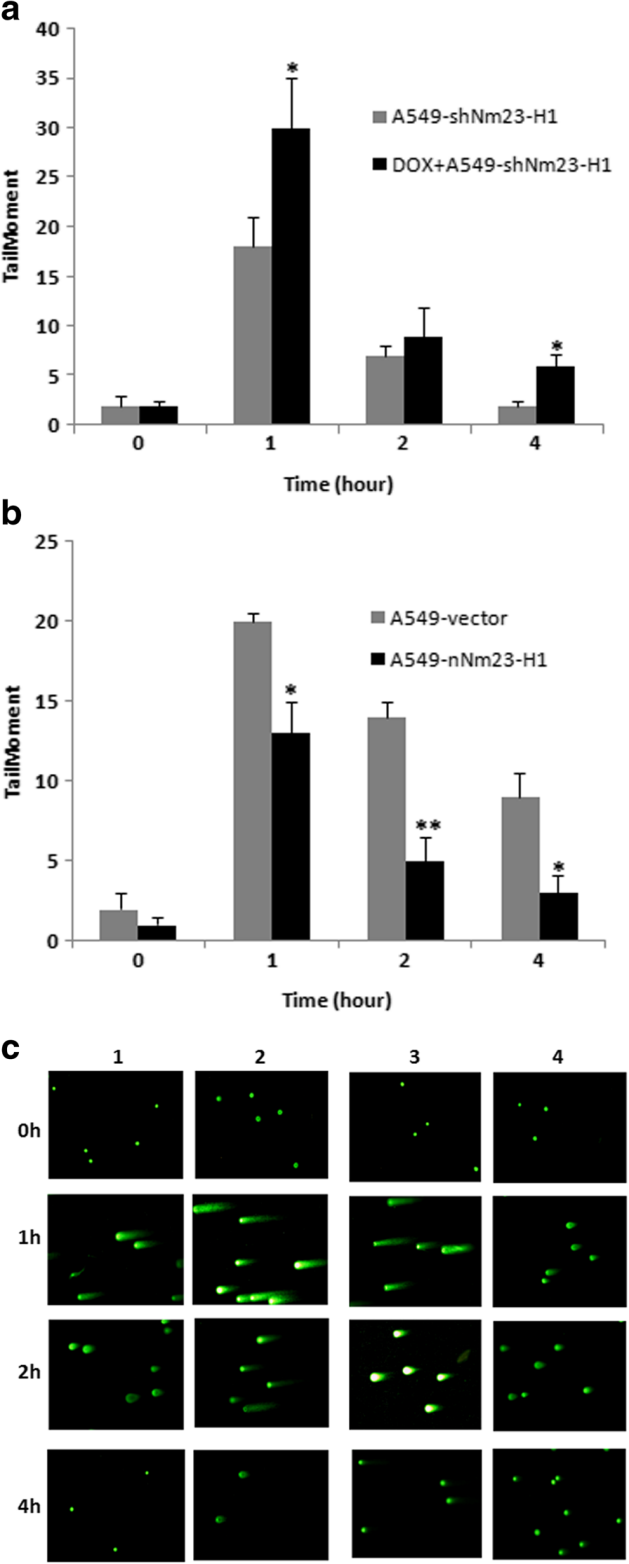
Quantification of DNA damage using Comet assay. All the cells were collected at 0 h, 1 h, 2 h, and 4 h after irradiation with 8 Gy X-rays. DNA damage was evaluated as the tail moment, combining comet tail length and the proportion of DNA migrating into the tail. **a** Nm23-H1-low-expressing group. **b** Nm23-H1-over expressing group. **c** The tail moment image of both groups. 1. A549-shNm23-H1; 2. DOX+ A549-shNm23-H1; 3. A549-vector; 4. A549-nNm23-H1 (*: *p* < 0.05; **: *p* < 0.01)

Colony formation assay showed that there was no significant difference between Nm23-H1 low-expressing cells (A549-shNm23-H1) and the control group when the radiation dose was 2Gy and 4Gy. However, when administered with the dose of 6Gy and 8Gy, there were significant difference between these two groups (*t* = 2.913, *p* = 0.044; *t* = 2.996, *p* = 0.040). These data suggested that the suppression of Nm23-H1 resulted in increased sensitivity to high dose of X-ray, and thus the radiation dose of 8Gy was used as the dose for the follow-up experiment (Fig. 2).Quantification of DNA damage in all cells using a Comet assay (measured in olive tail moment(OTM)) showed that 1 and 4 h after 8 Gy X-ray irradiation, Nm23-H1 low-expressing cells displayed significantly greater DNA damage compared with control cells (*t* = 3.919, *p* = 0.017; *t* = 3.674, *p* = 0.021), as measured by the tail moment. In contrast, Nm23-H1-overexpressing cells (A549-nNm23-H1) had lower DNA damage compared with control cells 1, 2, and 4 h after irradiation (*t* = 4.382, *p* = 0.012; *t* = 4.899, *p* = 0.008; *t* = 3.873, *p* = 0.018;) (Fig. 3).

**Fig. 4:**
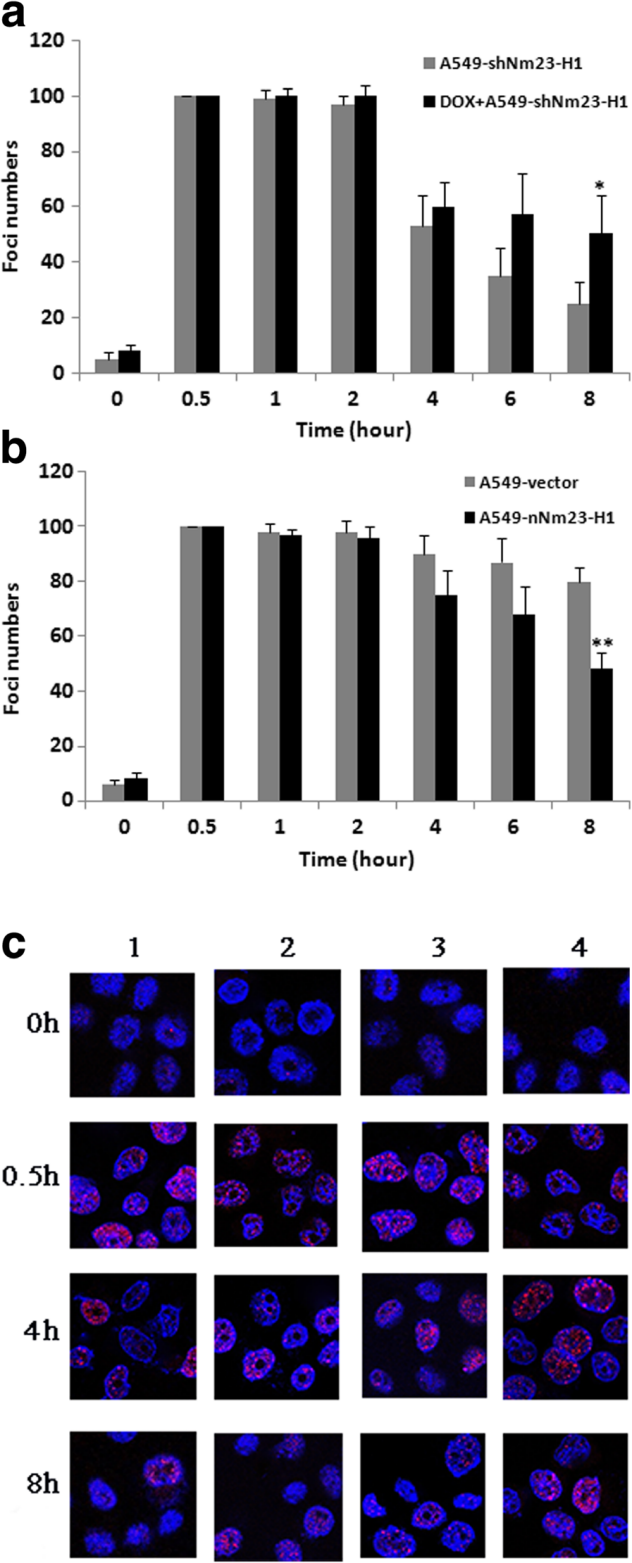
Quantification of DNA damage using γ-H2AX foci numbers. All the cells were collected at 0 h, 0.5 h, 1 h, 2 h, 4 h, 6 h and 8 h after irradiation with 8 Gy X-rays. DNA damage was evaluated as γ-H2AX foci numbers. **a** Nm23-H1-low-expressing group. **b** Nm23-H1-over expressing group. **c** The γ-H2AX foci image of both groups. The γ-H2AX protein is stained with red fluorescent and the nucleus is stained with blue fluorescent. 1. A549-shNm23-H1; 2. DOX+ A549-shNm23-H1; 3. A549-nNm23-H1; 4. A549-vector. (*: *p* < 0.05; **: *p* < 0.01)

As a second measure of the cellular response to DNA damage, γ-H2AX foci numbers were also assessed. Thirty minutes after X-ray irradiation, a marked increase in γ-H2AX-positive cells was observed in all cell lines. Eight hours after irradiation, only 50% of DSBs were repaired in the Nm23-H1-low expressing cells, compared to 70% in control cells (t = 3.873, *p* = 0.018). In contrast, twice as many DSBs were repaired in the Nm23-H1-overexpressing cells compared to control cells (*t* = 7.097, *p* = 0.002), consistent with the Comet assay results (Fig. 4).

### Nm23-H1 activates the phosphorylation of checkpoint pathway proteins

Checkpoint signaling is activated in response to incomplete DNA replication and DNA damage induced by both internal and external sources. Active checkpoints prevent further progression through the cell cycle, allowing time for DNA repair. The ATM protein kinase (ATM)/checkpoint kinase 2 (Chk2) module is activated by DSBs, leading to ATM- and Chk2-mediated phosphorylation of p53 at multiple residues. In turn, p53 triggers the transcription of the potent cyclin-dependent kinase inhibitor p21, which is crucial for the G1/S checkpoint. Thus ATM/Chk2/p53 is a key pathway inducing cell cycle arrest.

**Fig. 5:**
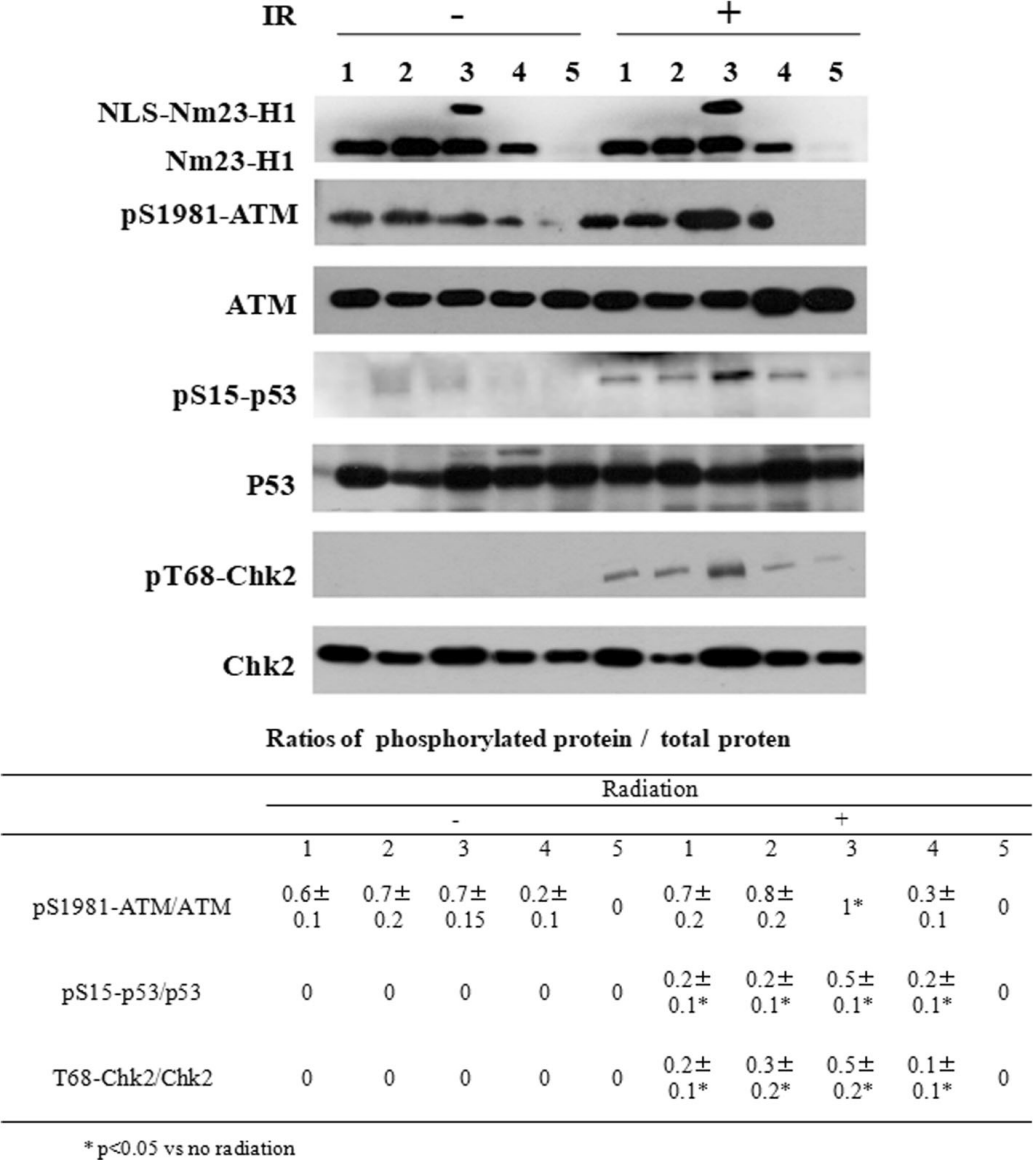
Phosphorylation of checkpoint pathway related proteins detected by Western blot. Cells were collected at 0 h and 8 h after irradiation with 8 Gy X-rays. Checkpoint pathway related proteins were detected by Western blot. Note that radiation treatment increased the overall levels of pT68-Chk2, pS15-p53 and pS1981-ATM and the Nm23-H1 level also related to the amount of the phosphorylated protein. 1 A549; 2 A549-vector; 3 A549-nNm23-H1; 4 A549-shNm23-H1; 5 DOX+ A549-shNm23-H1. (**: *p* < 0.01)

Cells were collected at 0 h and 8 h after irradiation. Western blot analysis showed that the levels of total ATM, p53, and Chk2 proteins were not affected by the Nm23-H1 level or irradiation. Also, although a weak pS1981-ATM signal was detected before irradiation, pS15-p53 and pT68-Chk2 phosphorylation was not detected in either the Nm23-H1 low-expressing or high-expressing groups before irradiation. However, phosphorylation of all the above mentioned sites was detected after irradiation, and the phosphorylation levels depended on the Nm23-H1 level. In Nm23-H1 overexpressing cells, pS1981-ATM, pS15-p53, and pT68-Chk2 phosphorylation was significantly higher, while in Nm23-H1 knockdown cells, phosphorylation at these sites was significantly inhibited. These data suggest that Nm23-H1 can activate the phosphorylation of checkpoint-related proteins and may participate in checkpoint activation during DSBR (Fig. 5).

### Nm23-H1 interacts with the MRN complex

The MRN complex, which includes NBS1, RAD50, and MRE11, is a major DSB sensor. It plays a special and central role in DSBR, restarting the DNA replication fork and signaling to cell cycle checkpoints [13]. We performed coimmunoprecipitation to detect whether Nm23-H1 interacted with the MRN complex in A549-nNm23-H1 cells.

**Fig. 6:**
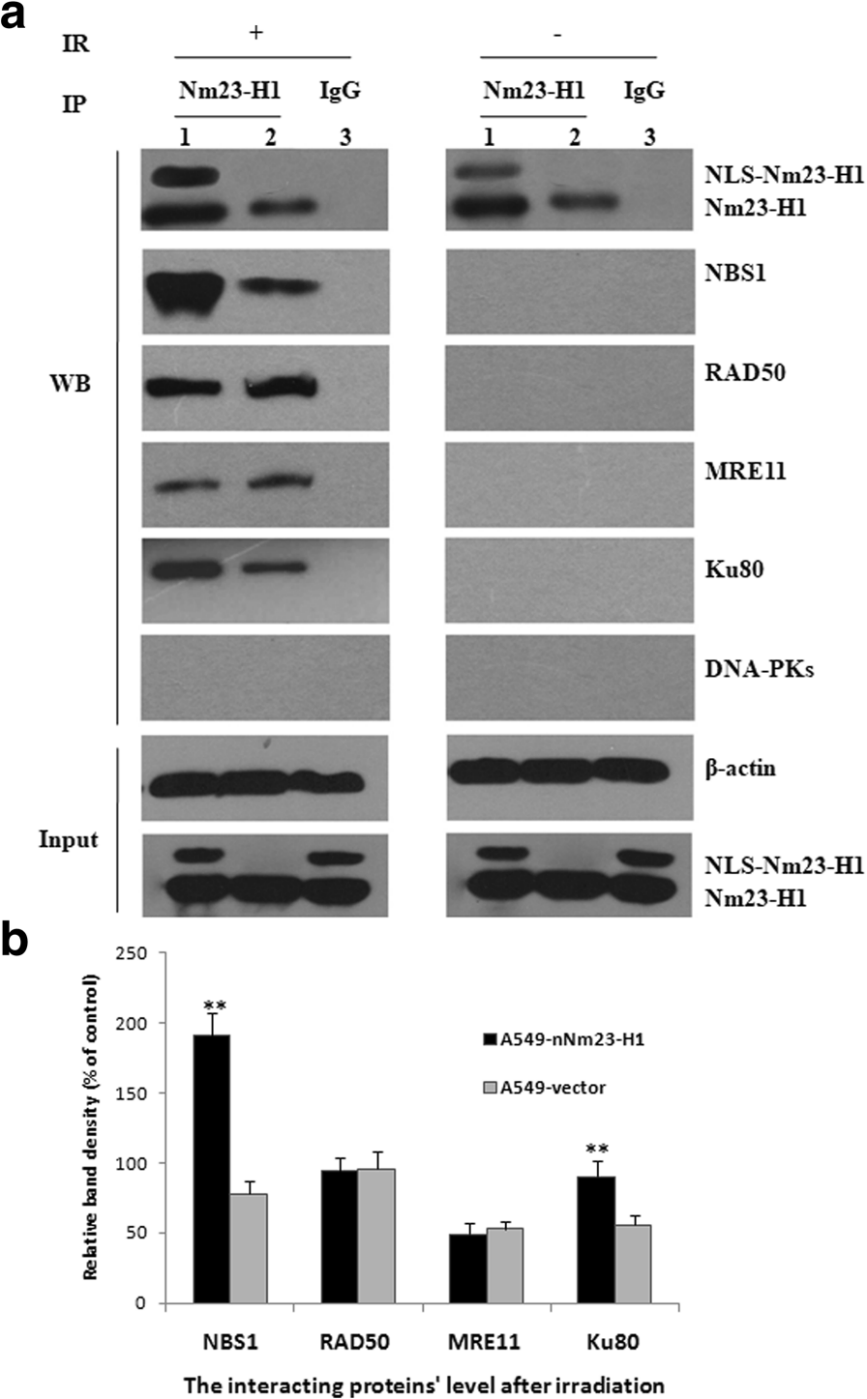
Proteins interacted with Nm23-H1 detected by co-immunoprecipitation. Cells were collected at 0 h and 8 h after irradiation with 8 Gy X-rays. Nm23-H1 antibody and IgG (as control) was used for pull the entire protein complex out of solution and then the binding proteins was detected by Western blot. 1 A549-nNm23-H1; 2 A549-vector; 3 A549-nNm23-H1. IR: ionizing radiation; IP: immunoprecipitation; WB: Western blot. (**: *p* < 0.01)

Cells were collected at 0 h and 8 h after irradiation. Before irradiation, no interactions between Nm23-H1 and NBS1, RAD50, or MRE11 were detected; however, interactions were observed after irradiation. Moreover, the NBS1 level was much higher in Nm23-H1 overexpressing cells than in control cells (*t* = 14.462, *p* < 0.001), suggesting that Nm23-H1 interacts with the MRN complex, potentially by binding NBS1 (Fig. 6).

### Nm23-H1 may promote DSB repair through non-homologous end joining (NHEJ)

In mammalian cells, irradiation-induced DSBs are repaired by two major pathways: NHEJ and homologous recombination (HR) [14], with the majority of repairs mediated by NHEJ [15]. Thus we tested whether Nm23-H1 interacted with the NHEJ-related proteins X-ray repair cross-complementing 5 (XRCC5, also known as Ku80), and the catalytic polypeptide of DNA-activated protein kinase (PRKDC, also known as DNA-PKcs) by coimmunoprecipitation in A549-nNm23-H1 cells.

Cells were collected 0 and 8 h after irradiation. Before irradiation, no interactions between Nm23-H1 and Ku80 and DNA-PKcs were detected. After irradiation, an interaction between Ku80 and Nm23-H1 was observed. Moreover, the Ku80 level was higher in the Nm23-H1 overexpressing cells compared to control cells (*t* = 5.347, *p* = 0.006). No interaction between DNA-PKcs and Nm23-H1 was observed in this experiment. As Ku80 is the keystone of the NHEJ pathway, we speculate that Nm23-H1 promotion of DSBR may occur through interaction with Ku80. However, more binding experiments are needed to verify this result (Fig. 6).

## Discussion

**Fig. 7:**
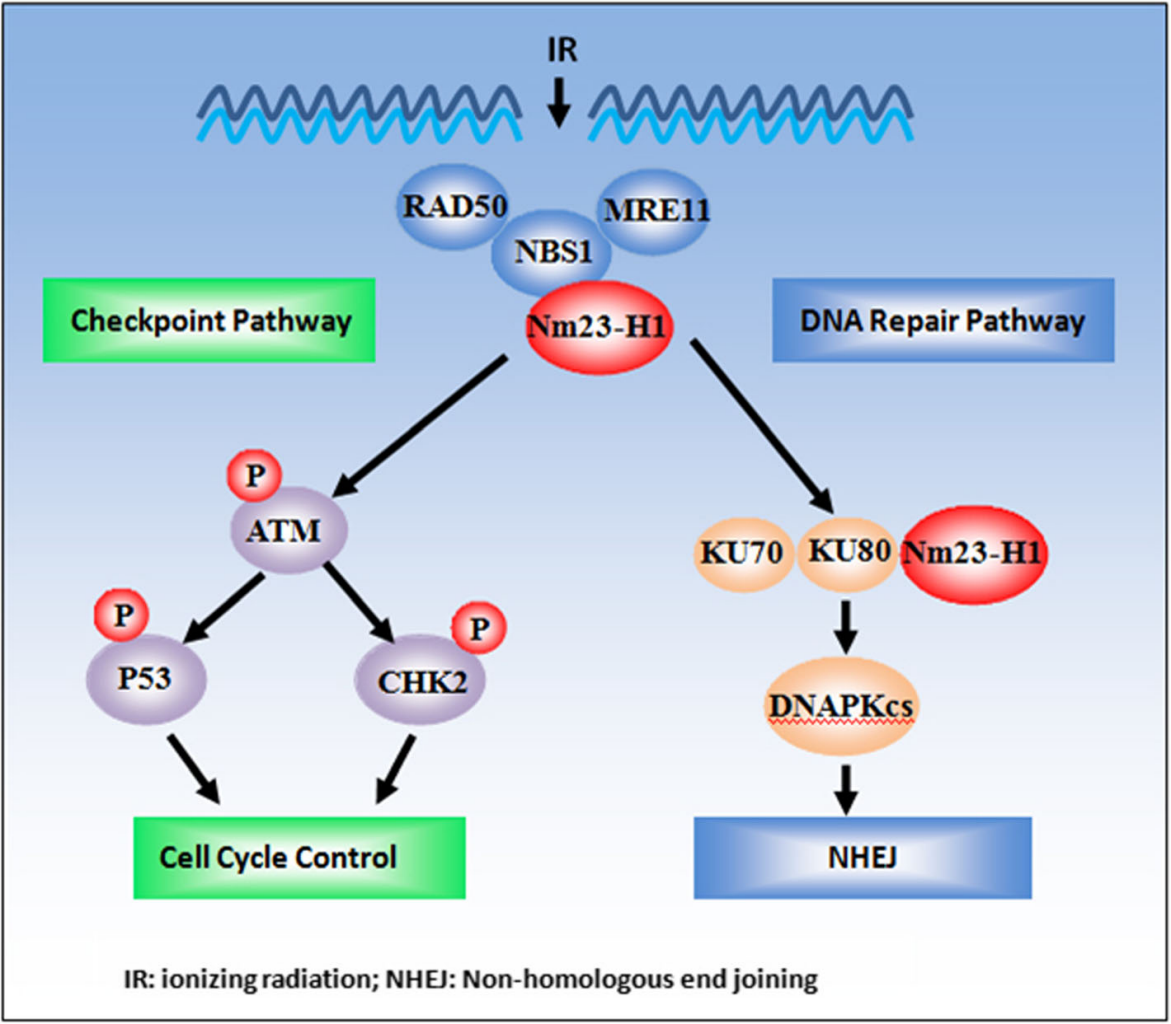
The mechanism of Nm23-H1 participating in multiple steps of DSBR pathway. Nm23-H1 involved in two pathways, which was cell-cycle checkpoint signaling pathway (ATM, Chk2 and p53 phosphorylated signaling pathway) and DNA repair pathway (interaction with Ku80). Moreover, Nm23-H1 also interacted with MRN complex. MRN complex functioned as the recognition and stabilization of DSBs and was the part of the DSB-sensing machinery. Thus the interaction of Nm23-H1 with MRN complex may promote the signaling of above two pathways

DSBs, the most lethal form of DNA damage, are introduced by exogenous agents such as ionizing radiation (IR) and certain drugs (including topoisomerase poisons and radiomimetics), and by cellular processes such as V(D)J recombination, class switch recombination, stalled replication forks, and the generation of reactive oxygen species [14, 16]. In this study, we focused on the role of Nm23-H1 in the repair of IR-induced DSBs. Kaetzel et al. [17] observed that Nm23-H1 is recruited rapidly (within 30 min) to endonuclease I-PpoI-catalyzed DSBs, which suggested a novel role for NM23-H1 in DSBR. In this study, we found that Nm23-H1 participated in the repair of X-ray-induced DSBs, affecting both cell-cycle checkpoint signaling (by activating ATM, CHK2 and p53 phosphorylation) and DNA repair (by interacting with Ku80) pathways. Moreover, Nm23-H1 also interacted with the MRN complex, which functions in the recognition and stabilization of DSBs as part of the DSB-sensing machinery. Thus the interaction betweenNm23-H1 and the MRN complex may promote the activation of the abovementioned pathways. Taken together, the data indicate that Nm23-H1 participates in multiple steps of DSBR (Fig. 7), and may play an extensive role in its promotion.

In 2002, Ma et al. [18] reported that Nm23-H1 possesses3’-5′ exonuclease activity, suggesting potential roles in DNA repair [19]. Since then, more and more studies have confirmed its involvement. However, the role of the 3′-5′ exonuclease activity remains to be elucidated. Jarrett reported that the NDPK kinase activity of NM23-H1, but not its 3′-5′ exonuclease function, promotes NER, suggesting that the NDPK activity may play a key role in DNA repair. The NDPK activity is important for maintaining balanced nucleotide pools, which mainly affects DNA synthesis and protein phosphorylation. NDPK-deficient strains lead to increases in dCTP and dGTP and decreased dATP [20]. In recent years, studies have confirmed that enzymes involved in nucleotidebalance are related to DSBR [21, 22]. For example, thymidylate kinase and nucleotide reductase (RNR) are involved in DSBR through their effects on dNTPs [21, 22]. Taso et al. reported that disruption of interactions between Nm23-H3, Tip60, and RNR suppressed DSBR in serum-deprived cells, as RNR-mediated catalysis produces dNDPs, and subsequent dNTP formation requires NDPK function [23]. Thus we speculated that the NDPK kinase activity of Nm23-H1 was also involved in DSBR pathway.

In mammalian cells, NHEJ and HR are responsible for the repair of IR-induced DSBs [14]. HDR performs high-accuracy repair that requires an undamaged sister chromatid to act as a template, and functions only after DNA replication [14, 15]. Conversely, NHEJ is active throughout the cell cycle [24] and therefore repairs the majority of IR-induced DSBs [25]. However, IR results in complex DNA ends that are often contain nonligatable end groups and other damage, which must be processed prior to NHEJ-mediated ligation. Werner syndrome RecQ-like helicase, which has 3′-5′ exonuclease activity, is reported to serve this role through an interaction with Ku70/80 [26–28]. In this study, coimmunoprecipitation experiments revealed an interaction between Nm23-H1 and Ku80, suggesting a potential role for Nm23-H1, which also has 3′-5′ exonuclease activity in NHEJ.

This study also found that Nm23-H1 was able to interact with the MRN complex after radiation, and that NBS1 expression increased significantly with increased Nm23-H1 expression. MRE11 and RAD50 are mainly present in the cytoplasm. When DSBs occur, these proteins rapidly translocate into the nucleus, a process mediated by NBS1 [29]. The mechanism of Nm23-H1 translocation into nucleus remains unclear, but the results suggest that its translocation may also be mediated by NBS1.

## Conclusions

In summary, we found that Nm23-H1 participated in the repair of X-ray-induced DSBs. Nm23-H1 impacted both cell-cycle checkpoint signaling, by increasing ATM, Chk2, and p53 phosphorylation, and DNA repair signaling, by interacting with components of the NHEJ pathway. However, there two issues remain unsolved: the first is whether Nm23-H1 also participates in HR. As the HR pathway requires DNA synthesis and thus abundant synthesis precursors (dNTPs) [30], the NDPK kinase activity of Nm23-H1 could be involved in the HR pathway. Secondly, the results do not directly implicate Nm23-H1 in NHEJ. These questions will be explored in future studies.

## Abbreviations

BER: Base excision repair
DOX: Doxycycline
DSBR: Double strand breaks repair
DSBs: Double strand breaks
HDR: Homology directed repair
IP: Immunoprecipitation
IR: Ionizing radiation
NDPK: Nucleoside diphosphate kinase
NER: Nucleotide excision repair
NHEJ: Non-homologous end joining
NLS: Nuclear located sequence
